# Gender Control of Mouse Embryos by Activation of TLR7/8 on X Sperm via Ligands dsRNA-40 and dsRNA-DR

**DOI:** 10.3390/molecules29010262

**Published:** 2024-01-04

**Authors:** Yunfei Hou, Jingfeng Peng, Linjun Hong, Zhenfang Wu, Enqin Zheng, Zicong Li

**Affiliations:** 1 National Engineering Research Center for Breeding Swine Industry, South China Agricultural University, Guangzhou 510642, China; hyf2021@stu.scau.edu.cn (Y.H.); 20231024011@stu.scau.edu.cn (J.P.); linjun.hong@scau.edu.cn (L.H.); wzf@scau.edu.cn (Z.W.); 2 State Key Laboratory of Swine and Poultry Breeding Industry, South China Agricultural University, Guangzhou 510642, China; 3 National and Local Joint Engineering Research Center for Livestock and Poultry Breeding Industry, South China Agricultural University, Guangzhou 510642, China; 4 Department of Animal Genetics, Breeding and Reproduction, College of Animal Science, South China Agricultural University, Guangzhou 510642, China; 5 Guangdong Provincial Key Laboratory of Agro-Animal Genomics and Molecular Breeding, South China Agricultural University, Guangzhou 510642, China; 6 Guangdong Provincial Laboratory of Lingnan Modern Agricultural Science and Technology, Guangzhou 510642, China

**Keywords:** gender control, sex ratio, dsRNA, TLR7/8, sperm separation

## Abstract

Gender control technologies are promising for enhancing the production efficiency of the farm animal industry, and preventing sex-linked hereditary diseases in humans. It has been shown that the X sperm of mammalian animals specifically expresses X-chromosome-derived toll-like receptor 7/8 (TLR7/8), and the activation of TLR7/8 on the X sperm by their agonist, R848, can separate X and Y sperm via the specific inhibition of X sperm motility. The use of R848-preselected sperm for fertilization resulted in sex-ratio-skewed embryos or offspring. In this study, we aimed to investigate whether two other TLR7/8 ligands, double-stranded RNA-40 (dsRNA-40) and double-stranded RNA-DR (dsRNA-DR), are also effective in the separation of mouse X and Y sperm and the subsequent generation of gender-ratio-skewed in vitro fertilization (IVF) embryos. Our results indicated that cholesterol modification significantly enhances the transfection of dsRNA-40 and dsRNA-DR into sperm cells. dsRNA-40 and dsRNA-DR incubation with mouse sperm could separate X and Y sperm by the specific suppression of X sperm motility by decreasing its ATP level and mitochondrial activity. The use of a dsRNA-40- or dsRNA-DR-preselected upper layer of sperm, which predominantly contains high-motility Y sperm, for IVF caused a male-biased sex ratio shift in resulting embryos (with 65.90–74.93% of embryos being male). This study develops a simple new method for the efficient separation of mammalian X and Y sperm, enabling the selective production of male or female progenies.

## 1. Introduction

In animal husbandry, the gender ratio is an important factor that can affect production efficiency. For example, in the dairy and poultry egg production industry, a higher female-to-male sex ratio is favorable for boosting milk yield and egg production [1,2]. Selective production of male or female breeding stock is beneficial for optimizing the reproductive potential of the parent population [3]. In humans, some hereditary diseases are transmitted via a gender-associated pattern [4,5]. Therefore, developing technologies for preselecting offspring sex has significant practical value in enhancing the production efficiency of the farm animal industry and reducing the transmission of sex-linked genetic diseases in humans.

At present, mammalian animal gender control can be achieved by flow-cytometry-based X/Y sperm separation and gene editing. Flow cytometry distinguishes X and Y sperm according to sex chromosome DNA content [6]. This method can preselect progeny sex with an accuracy of approximately 90% in farm animals like cattle, horses, sheep, and pigs [7,8,9,10,11,12]. However, it requires specialized equipment and expertise, with costs directly proportional to the number of separated sperm. This limitation makes it unsuitable for animals requiring a large dose of sperm for artificial insemination, such as pigs [13]. CRISPR/Cas9-mediated gene editing is a new, emerging technique for sex control. In 2019, Yosef et al. [14] demonstrated a CRISPR/Cas9 system for biasing mouse offspring ratios via the inactivation of developmental vital genes, specifically in male embryos. Douglas et al. [15] employed a synthetic CRISPR-Cas9 approach to producing a single-sex litter, albeit with a 30–40% reduction in litter size, by the specific elimination of male or female mouse embryos. In 2022, Bai [16] expressed a CRISPR/Cas9 system during mouse spermatogenesis to target the Y chromosome in Y sperm, generating female-biased progenies. Although these gene-editing-based technologies are promising for gender control, their commercial viability is limited by ethical and biosafety concerns related to genetic modifications. Hence, new techniques for animal gender control need to be developed.

In 2019, Umehara et al. [17] conducted a groundbreaking study, outlining a new method for the in vitro separation of X and Y sperm in mice using a TLR7/8 agonist, R848. Their research demonstrated that the activation of X-sperm-specific receptors TLR7/8 by the ligand R848 affected the glycolytic pathway and citric acid cycle, leading to reduced ATP levels and subsequent motility suppression. Y sperm, lacking TLR7/8, remained unaffected in terms of motility. This difference in X/Y sperm motility enabled their separation. Umehara later applied this system to separate frozen cattle semen, with similar results [18], offering a fresh perspective on animal sex control technology. In 2021, Chinese scientists led by Ren [19] applied this approach to treat goat sperm with R848, resulting in successful artificial insemination that produced sexually oriented young. Their research elucidated that R848 treatment inhibited X sperm motility in goats through the TLR7/8 signaling pathway, affecting X sperm ATP levels via the GSK3α/β–hexokinase pathway. In 2022, Huang [20] combined R848 with an alkaline semen diluent, achieving the successful isolation and enrichment of goat X sperm. This sex control technique, relying on the R848 activation of X sperm TLR7/8, was successfully validated in several mammals, including mice, cattle, and sheep, confirming the robustness of this sex control technology [17,18,19,20,21].

R848 is highly stable and can induce a long-term immune response [22]. It may cause unwanted side effects when used to treat sperm. In addition to R848, double-stranded (ds) RNA-40 and dsRNA-DR also serve as TLR7/8 ligands [23,24,25,26,27], and both dsRNA-40 and dsRNA-DR have the potential to activate TLR7 and TLR8 (TLR7/8). However, whether these ligands can specifically inhibit X sperm motility and can be employed for sex control are unknown. In this study, we investigated the effects of dsRNA-40 and dsRNA-DR on mouse sperm motility, mitochondrial membrane potential, ATP levels, the proportion of X/Y sperm in the upper semen layer, and the sex ratio of embryos generated by in vitro fertilization with dsRNA-treated semen. The findings from this study contribute to the development of novel methods for animal sperm separation and sex control.

## 2. Results

### 2.1. Efficient Transfection of Sperm with Cholesterol-Modified dsRNA-40 and dsRNA-DR

**Figure 1:**
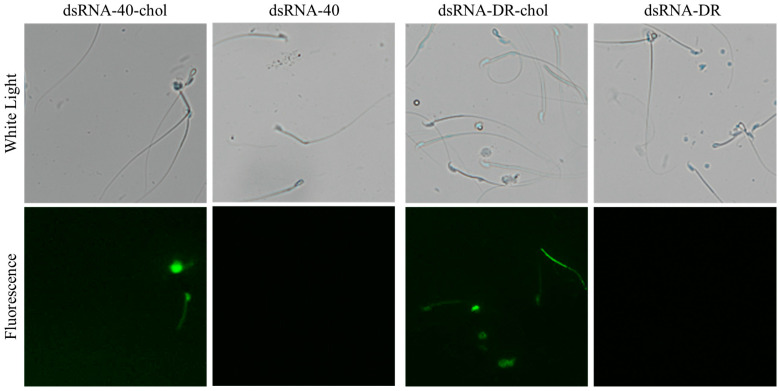
Efficient transfection of sperm with cholesterol-modified dsRNA-40 and dsRNA-DR.

TLR7/8 is usually located in the endosome inside eukaryotic cells [28,29,30]. Cholesterol modification has been shown to enhance the transfection of dsRNA into eukaryotic cells [31,32,33]. To facilitate the smooth entry of dsRNA into sperm to bind to TLR7/8, we used cholesterol-modified dsRNAs labeled with FAM fluorophore to incubate with mouse sperm (see Figure 1). Under fluorescence microscopy, green fluorescence was observed in sperm transfected with cholesterol-modified dsRNA-40 and dsRNA-DR, but not in sperm incubated with dsRNAs lacking cholesterol modification. These results suggest that cholesterol-modified dsRNA can efficiently penetrate sperm.

### 2.2. Determination of Incubation Concentration and Time of dsRNA-40 and dsRNA-DR for Treatment of Mouse Sperm

**Figure 2:**
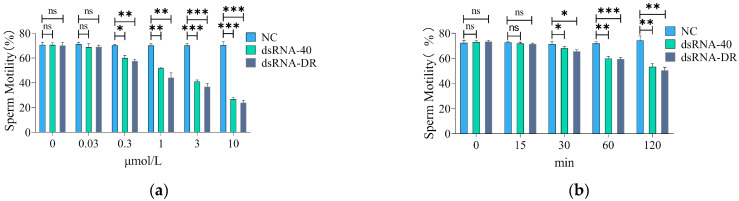
Determination of incubation concentration and time of dsRNA-40 and dsRNA-DR for treatment of mouse sperm. (**a**) Sperm motility was assessed following a 60 min incubation with varying concentrations of dsRNA-40 or dsRNA-DR; (**b**) sperm motility after incubation with 0.3 μM dsRNA-40 or dsRNA-DR for different times. Values represent the mean of three replicates ± Standard Error of the Mean (SEM). NC denotes non-sense double-stranded RNA as control. ns denotes no statistically significant difference compared to control group, *p* > 0.05; * denotes a significant difference compared to the control group, *p* < 0.05; ** denotes a highly significant difference compared to the control group, *p* < 0.01; *** denotes an extremely significant difference compared to the control group, *p* < 0.001.

To investigate the impact of TLR7/8 agonists dsRNA-40 and dsRNA-DR on mouse sperm motility, various concentrations of dsRNA-40 or dsRNA-DR were incubated with sperm for 1 h, followed by the assessment of sperm motility. The results presented in Figure 2a demonstrate that treatment with 0.3, 1, 3 and 10 μM dsRNA-40 or dsRNA-DR led to a significant reduction in sperm motility, and this effect exhibited concentration dependence. These data suggest that if employing dsRNA-40 and dsRNA-DR as TLR7/8 ligands to specifically inhibit the motility of X sperm, a concentration of 0.3 μM or higher should be used. Taking into account the findings from Umehara’s study [17] that using 0.3 μM of R848, another TLR7/8 agonist, to incubate with mouse sperm is effective in separating X and Y sperm, we decided to utilize 0.3 μM dsRNA for the subsequent treatment of mouse sperm.

To further investigate the time required for 0.3 μM dsRNA treatment to alter sperm motility, mouse sperm were incubated with 0.3 μM dsRNA, and sperm motility was assessed at various timepoints. The results presented in Figure 2b demonstrate that treatment with 0.3 μM dsRNA-40 or dsRNA-DR for 30, 60, and 120 min significantly decreased sperm motility, and this effect displayed a time-dependent pattern. These findings imply that the treatment of mouse sperm with 0.3 μM dsRNA-40 or dsRNA-DR for 30 min may be effective in reducing X sperm motility. To avoid the detrimental effects caused by a longer treatment with dsRNA on sperm, we chose 30 min as the incubation time for subsequent dsRNA treatment of mouse sperm.

### 2.3. dsRNA-40 and dsRNA-DR Can Separate Mouse X and Y Sperm by Specific Inhibition of X Sperm Motility

The findings of Umehara [17] and Renfa [19] have shown that, after sperm treatment with R848, highly motile Y-bearing sperm are primarily located in the upper layer, while the lower layer predominantly contains slow-moving X-bearing sperm. To investigate whether the treatment of sperm with dsRNA-40 or dsRNA-DR can alter the ratio of X/Y sperm in the upper and lower layers, this study employed quantitative PCR based on the X-chromosome-specific gene Akap4 and the Y-chromosome-specific gene ddx3y to assess the percentage of Y and X sperm in the upper and lower layers. 

**Figure 3:**
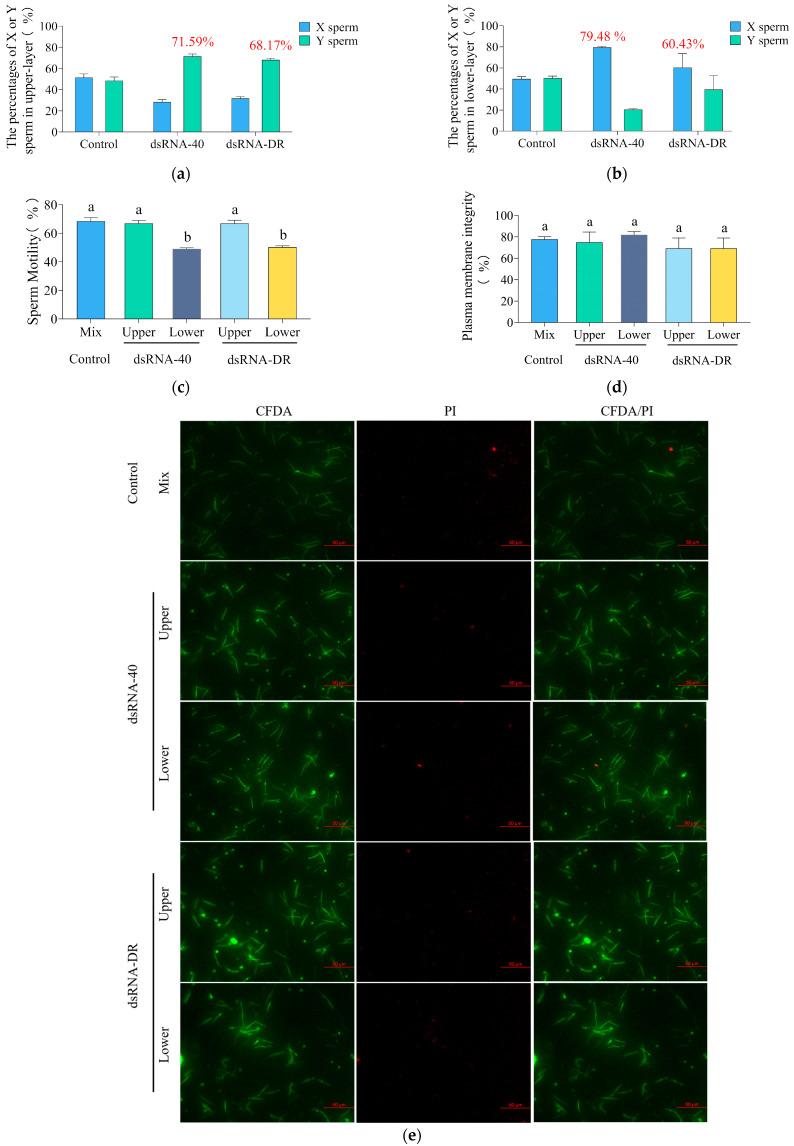
dsRNA-40 and dsRNA-DR can separate mouse X and Y sperm by the specific inhibition of X sperm motility. (**a**) The percentage of X and Y sperm in the upper layer after incubation with 0.3 μM dsRNA-40 or dsRNA-DR for 30 min; (**b**) the percentage of X and Y sperm in the lower layer after incubation with 0.3 μM dsRNA-40 or dsRNA-DR for 30 min; (**c**) comparison of motility in the upper and lower layers of sperm after incubation with 0.3 μM dsRNA-40 or dsRNA-DR for 30 min; (**d**) comparison of plasma membrane integrity of sperm in the upper and lower layers after incubation with 0.3 μM dsRNA-40 or dsRNA-DR for 30 min; (**e**) sperm plasma membrane integrity was analyzed by staining with 6-CFDA/PI. Different letters indicate statistically significant differences between groups, *p* < 0.05, while the same letters indicate no significant differences between groups, *p* > 0.05.

The results indicate that, in the 0.3 μM dsRNA-40 treatment group, the percentage of Y sperm in the upper layer was 71.59% ± 3.73% (Figure 3a), while the percentage of X sperm in the lower layer was 79.48% ± 1.44% (Figure 3b). This study employed the dsRNAs transfection of spermatozoa in 1 mL of HTF medium, distributed into 1.5 mL centrifuge tubes. The upper layer was defined as the top 350 μL of sperm resuspension in the centrifuge tubes, while the lower layer was designated as the bottom 350 μL of sperm resuspension, mouse sperm collection methods and a schematic are provided in Supplementary Material Figure S1. In the 0.3 μM dsRNA-DR treatment group, the percentage of Y sperm in the upper layer was 68.17% ± 2.72% (Figure 3a), and the percentage of X sperm in the lower layer was 60.43% ± 23.07% (Figure 3b). These findings suggest that the two tested dsRNAs can separate mouse X and Y sperm into two different layers. CASA analysis results indicate that the two dsRNA treatments significantly decreased the motility of the X-sperm-containing lower-layer sperm, but not the Y-sperm-containing upper-layer sperm, as compared to the control group (Figure 3c). The combined results of quantitative PCR and CASA analysis suggest that dsRNA-40 and dsRNA-DR can separate mouse X and Y sperm by the specific inhibition of X sperm motility, thereby leading to the upper layer containing predominantly high-motility Y sperm, while the lower layer predominantly consisting of slow-moving X sperm.

To further investigate whether the difference in sperm motility between the upper and lower layers is a result of sperm damage, we employed the 6-CFDA/PI assay kit to assess sperm plasma membrane integrity. The results in Figure 3d,e demonstrate that the plasma membrane integrity of both the upper- and lower-layer sperm is not significantly reduced compared to the control group. This finding suggests that dsRNA-40 or dsRNA-DR treatment has no significant impact on sperm plasma membrane integrity.

### 2.4. dsRNA-40 and dsRNA-DR Inhibit X Sperm Motility by Reducing ATP Level and Mitochondrial Activity

Sperm velocity is regulated by intracellular ATP levels, with mitochondria serving as a crucial energy source for sperm motility. To investigate whether the differences in sperm motility between the upper and lower layers result from variations in mitochondrial activity and ATP levels, we assessed the ATP levels and mitochondrial activity of sperm after treatment with dsRNA-40 or dsRNA-DR.

**Figure 4:**
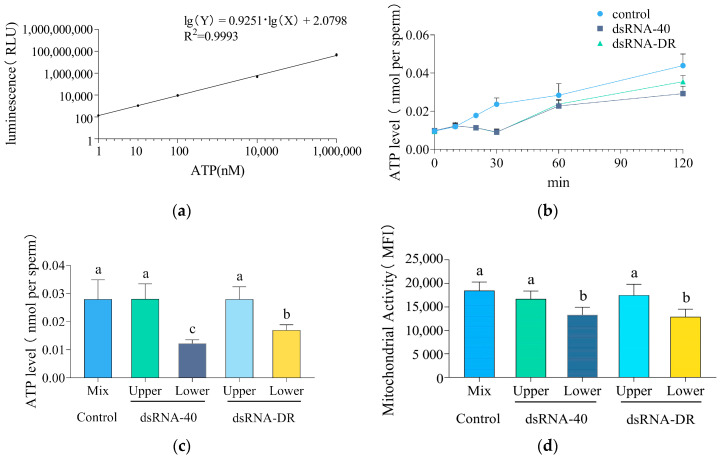
dsRNA-40 and dsRNA-DR inhibit X sperm motility by reducing ATP level and mitochondrial activity. (**a**) ATP standard curve with ATP concentrations of 1 mM, 10 μM, 100 nM, 10 nM, and 1 nM; (**b**) changes in sperm ATP levels following incubation with or without 0.3 μM dsRNA-40 or dsRNA-DR; (**c**) comparison of ATP levels between the upper and lower layer sperm after treatment with 0.3 μM dsRNA-40 or dsRNA-DR for 30 min; (**d**) comparison of mitochondrial activity between the upper and lower layer sperm after treatment with 0.3 μM dsRNA-40 or dsRNA-DR for 30 min. Different letters indicate statistically significant differences between groups, *p* < 0.05, while the same letters indicate no significant differences between groups, *p* > 0.05.

Sperm ATP levels were determined using the standard curve formula. The high linearity coefficient (R2) of the ATP standard curve, reaching 0.9993, indicates its remarkable reliability (Figure 4a). As depicted in Figure 4b, after incubation with two tested dsRNAs for more than 10 min, sperm ATP levels began to decline compared to the control group, and this decline trend persisted over time, indicating that both dsRNA-40 and dsRNA-DR could inhibit sperm ATP production.

Following treatment with 0.3 μM dsRNA-40 or dsRNA-DR for 30 min, a decrease in ATP levels was observed, primarily in the lower layer of sperm with reduced motility (Figure 4c). Moreover, compared to the control group, mitochondrial activity in the lower-layer sperm was significantly reduced (Figure 4d). These data confirm that dsRNA-40 and dsRNA-DR impact X sperm motility by reducing ATP levels and mitochondrial activity.

### 2.5. Production of Embryos with a Male-Biased Sex Ratio by Using Y Sperm Preselected with dsRNA-40 and dsRNA-DR Treatment for IVF

**Figure 5:**
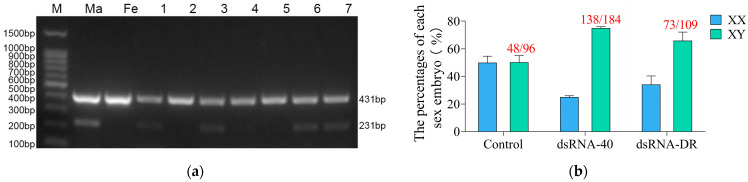
Production of embryos with a male-biased sex ratio by using Y sperm preselected with dsRNA-40 and dsRNA-DR treatment for IVF. (**a**) Dual PCR-based sex determination of in vitro-fertilized embryos (blastocysts) generated by the upper layer of sperm separated with 0.3 μM dsRNA-40 or dsRNA-DR treatment for 30 min. To determine the gender of individual mouse blastocysts and to prevent false negatives due to sample loss, a dual PCR approach was employed. The upper band (431 bp) indicates the presence of an autosome-linked gene, while the lower band (231 bp) signifies the presence of a Y-chromosome-linked gene. The upper band is used to verify the success of PCR, and the lower band is used to determine the sexuality of tested IVF embryos. The male control group successfully amplified a band corresponding to the Y-chromosome-specific gene ddx3y. On the other hand, the female control group failed to produce an amplification band of such an amount. These findings demonstrate the accuracy of the dual PCR method for embryo sex identification. M, male control. F, female control. #1, #3, #6 and #7 were identified as male embryos, while #2, #4 and #5 were identified as female embryos; (**b**) the gender ratio of IVF embryos produced by the upper layer of sperm separated with 0.3 μM dsRNA-40 or dsRNA-DR treatment for 30 min. Three experiments were conducted in each group. The total number of identified male embryos and the total number of analyzed IVF embryos are shown at the top of each group’s column.

In this study, we developed a reliable method for determining the sex of individual blastocysts using a dual-PCR-based method (Figure 5a). To further substantiate that the upper layer of highly motile sperm predominantly consists of Y sperm, in vitro fertilization was conducted using the upper layer of mouse sperm separated by dsRNA treatment. The results displayed in Figure 5b show that when in vitro fertilization was carried out using the upper-layer mouse sperm preselected by dsRNA-40 or dsRNA-DR incubation, a total of 96, 184, and 109 embryos were obtained. Among these, male (XY) embryos accounted for 48 (50.17% ± 8.42%), 138 (74.93% ± 1.68%), and 73 (65.90% ± 10.70%), while female (XX) embryos accounted for 48 (49.83% ± 8.42%), 46 (25.06% ± 1.68%), and 36 (34.10% ± 10.70%), in the control, dsRNA-40 and dsRNA-DR groups, respectively. The production of predominantly male IVF embryos from the upper-layer sperm indicates the successful separation of mouse X and Y sperm by dsRNA treatment.

## 3. Discussion

TLR7/8 are toll-like receptors (TLRs) that perceive nucleic acids. They are usually located in the lysosomes within the cells. Segregated TLR7/8 TLR effectively prevents their contact with host DNA or RNA, thus avoiding the activation of innate immune responses. This also determines that extracellular RNA or DNA need to be transported into cells for the binding of nucleic-acid-sensing TLRs [30]. The TLR7/8 agonist R848 is a small-molecule compound, which can easily penetrate the cell membrane to activate TLR7/8 [34,35]. However, without cholesterol modifications, dsRNA-40 and dsRNA-DR could not pass through the sperm cell membrane. To facilitate the successful intracellular activation of TLR7/8 by dsRNA-40 and dsRNA-DR, we introduced cholesterol modifications at the 3′ end of the dsRNAs. While significantly increasing the transfection efficiency of dsRNAs, cholesterol modifications seem not to alter the binding function of dsRNA-40 and dsRNA-DR to TLR7/8, as the motility of TLR7/8-expressing X sperm was specifically inhibited by dsRNA-40 and dsRNA-DR. A similar result was also reported in another study [36].

Previous studies [17,19] indicated that the binding of R848 agonist to receptors TLR7/8 decreases ATP production and mitochondrial function by regulating the downstream GSK3 α/β-hexokinase pathway specifically in X sperm, without impacting Y sperm. We found in this study that the incubation of mouse sperm with TLR7/8 ligands dsRNA-40 and dsRNA-DR also reduces ATP level and mitochondrial activity in X sperm. This suggests that dsRNA-40 and dsRNA-DR also act through the downstream GSK3 α/β-hexokinase pathway of TLR7/8 to suppress the motility of X sperm.

In this study, the treatment of mouse sperm with 0.3 µM dsRNA-40 or dsRNA-DR for 30 min resulted in the upper layer of sperm carrying 68.17–71.59% of Y sperm. However, the incubation of mouse sperm with 0.3 µM R848 for 30 min resulted in 91.0% of sperm in the upper layer being Y sperm [17]. Furthermore, the use of dsRNA-40- or dsRNA-DR-preselected upper layer of sperm for IVF-generated mouse embryos resulted in a male ratio of 65.9–74.93%, while the use of R848-separated upper layer of sperm for IVF-produced mouse embryos resulted in a male ratio of 89.3% [17]. The effectiveness of the two investigated dsRNAs on separating mouse X and Y sperm seems lower than that of R848. This difference could be related to the difference in TLR7/8 binding activity between dsRNAs and R848 because dsRNAs are weaker TLR7/8 agonists than R848 [37,38,39]. The difference in X and Y sperm separation efficiency between dsRNAs and R848 also could be due to their difference in half-life, since dsRNAs are easily degraded [40,41], whereas R848 is a stable compound [22]. However, the efficiency of dsRNA-40 or dsRNA-DR in separating mouse X and Y sperm could be further improved by optimizing the incubation concentration and time with mouse sperm.

When choosing TLR7/8 agonists to separate X and Y sperm, the cost of the agonists is also a critical factor that should be considered, in addition to the effectiveness. Some company websites (InvivoGen Company, San Diego, CA, USA, https://www.invivogen.com/r848, accessed on 1 November 2023; Miltenyi Biotec, Bergisch Gladbach, Germany, https://www.miltenyibiotec.com/CN-en/products/r848-resiquimod.html, accessed on 1 November 2023) indicate that the price of 500 μg (1.43 μmoles) of R848 is 2087 CNY, which is approximately equivalent to 560 OD of dsRNA. The synthesis of this amount of dsRNA by some company (GenePhaema Company, Suzhou, China, website: http://www.genepharma.cn/, accessed on 1 November 2023) costs about 9000 CNY. Although the dsRNA price looks higher than R848, it is a specialized custom product, and its cost is expected to significantly decrease with large-scale synthesis.

Although we demonstrated that tge dsRNA-40 and dsRNA-DR treatment of mouse sperm is feasible in the gender control of embryos, whether they can be employed to produce offspring with a skewed sex ratio is unknown. In addition, due to the lack of specialized equipment and knowledge of mouse embryo transfer technique in our laboratory, we did not use this technique to further validate the role of dsRNAs, which is a major drawback of this study. Since artificial insemination techniques are not particularly mature in mice, we did not use dsRNA-treated mouse sperm for artificial insemination to produce progeny in the present study. Nevertheless, artificial insemination techniques are easily performed and usually have a high success rate in livestock such as cattle and pig. Future studies can be conducted to test whether dsRNA-40 and dsRNA-DR are feasible in the sex ratio control of livestock.

## 4. Materials and Methods

### 4.1. Animals

Eight-week-old male ICR mice (Supplier: SPF (Beijing) Biotechnology Co., Ltd., Beijing, China, Catalog No. YD012) and eight-week-old female ICR mice (Supplier: SPF (Beijing) Biotechnology Co., Ltd., Catalog No. YD012) were utilized in this study. The use of experimental animals was approved by the Ethics Committee of the Experimental Animal Center of South China Agricultural University (License No.: SYXK-2019-0136).

### 4.2. Biochemical Materials

RNA was custom-designed and synthesized by Suzhou GenePhaema Co., Ltd. (Suzhou, China). The carboxyfluorescein (FAM) moiety was added at the 5′ end of dsRNA, and cholesterol modification was added at the 3′ end of dsRNA. dsRNA-40 sense (5′-3′): GCCCGUCUGUUGUGUGACUCTT; antisense (5′-3′): GAGUCACACAACAGACGGGCTT. DsRNA-DR sense (5′-3′): UGACCUUCAAUGUCCUUCAATT; antisense (5′-3′): UUGAAGGACAUUGAAGGUCATT. The qPCR primers were designed using Primer Premier 6.

Human chorionic gonadotropin hCG (Catalog No.: M2520) and pregnant mare serum gonadotropin PMSG (Catalog No.: M2620), HTF fertilization medium (Catalog No.: M1130), M2 culture medium (Catalog No.: M1250), KSOM embryo culture medium (Catalog No.: M1430), and hyaluronidase (Catalog No.: M2215) were all obtained from Nanjing Aibei Biotechnology Co., Ltd. (Nanjing, China).

PCR primers and the direct PCR amplification kit (B639289) were procured from Sangon Biotech Co., Ltd. (Shanghai, China).

PowerUp SYBR Green Master Mix (A25742) was obtained from Thermo Fisher Scientific (Shanghai, China).

The ATP cell viability assay kit (Catalog No.: 40210ES10) was sourced from Yisheng Biotechnology Co., Ltd. (Shanghai, China).

The mitochondrial membrane potential assay kit (TMRE) (Catalog No.: C2001S) was obtained from Shanghai Biyuntian Biotechnology Co., Ltd. (Shanghai, China).

All other materials not explicitly mentioned were purchased from Sigma-Aldrich China (Shanghai, China).

### 4.3. Sperm Transfection with Cholesterol-Modified dsRNAs

The mouse spermatozoa were co-incubated with dsRNA-40, dsRNA-DR with or without cholesterol modification, at a concentration of 0.3 μM for 10 min at 37 °C, in a 5% CO_2_ incubator. The fluorescent probe FAM (carboxyfluorescein) was used with an absorption wavelength of 492 nm and an emission wavelength of 518 nm. A fluorescence microscope was used to detect green fluorescence production in sperm.

### 4.4. Assessment of Sperm Motility Using the CASA System

Sperm were incubated at 37 °C in an HTF medium containing 0.3 μM of dsRNA-40 or dsRNA-DR for 30 min. Subsequently, the motility parameters of sperm in the upper and lower layers were assessed using a computer-assisted sperm analysis (CASA) system (HVIEW-SSAV600.8, Fuzhou Hongshiye Software Technology Co., Ltd., Fuzhou, China). Each experiment was repeated four times.

### 4.5. Measurement of Sperm Plasma Membrane and Mitochondrial Activity

The integrity of the plasma membrane of mouse sperm treated with dsRNA-40 or dsRNA-DR was assessed using the 6-carboxyfluorescein diacetate/propidium iodide (6-CFDA/PI) assay kit in HTF medium. Initially, 10 μL of 6-CFDA was gently mixed with the treated mouse sperm and incubated at 37 °C for 30 min. Subsequently, 5 μL of PI reagent was added, and incubation was continued for 10 min at 37 °C. Double-stained sperm (20 μL) were transferred to a microscope slide, covered with a coverslip, and immediately observed and counted at 200× magnification under a fluorescence microscope, ensuring that no fewer than 200 sperm were observed in each field of view. The 6-CFDA freely diffuses inside cells and is hydrolyzed by non-specific lipase within the cell to produce carboxyl fluorescein (CF). The fluorescence product accumulates only in cells with intact cell membranes and active esterase activity, while dead cells remain unstained.

To assess changes in mitochondrial membrane potential in mouse sperm following treatment with dsRNA-40 or dsRNA-DR, 10 μL of TMRE staining working solution was gently mixed with the treated mouse sperm and incubated for 30 min at 37 °C. Subsequently, centrifugation at 1000× *g* for 3 min was performed, followed by two washes. The measurements were carried out using a multi-mode microplate reader (BioTek Synergy H1, Agilent Technologies, Santa Clara, CA, USA). TMRE is an orange-red cationic fluorescent probe that permeates the cell membrane, accumulates in intact mitochondria, and decreases in depolarized or inactive mitochondria, resulting in reduced TMRE accumulation. The excitation/emission wavelengths were 550 nm/575 nm.

### 4.6. Determination of Sperm ATP Content

Sperm ATP content measurement was conducted using the ATP assay kit (Catalog No.: 40210ES10, Yeasen Biotechnology, Shanghai, China). Sperm were incubated in HTF medium for various durations (0, 10, 20, 30, 60, and 120 min), both with and without treatment with dsRNA-40 or dsRNA-DR. Equal aliquots of samples from the upper and lower layers (100 μL, diluted to 1 × 10^7^ sperm/mL) were added to a 96-well white polystyrene microplate, and 100 μL of the assay reagent was added to each well. The mixture was shaken at room temperature for 2 min to facilitate cell lysis and then left at room temperature for 10 min until stabilization. Chemical luminescence measurements were subsequently performed using a multi-mode microplate reader (BioTek Synergy H1, Agilent Technologies) with a detection wavelength of 560 nm.

An ATP standard curve was generated by adding various concentrations of ATP standard solutions (1 mM, 10 μM, 100 nM, 10 nM, 1 nM) to each well of the 96-well plate, with each well containing 100 μL of the standard solution. The ATP content was calculated based on the ATP standard curve to determine the relative vitality of the cells.

### 4.7. Measurement of the Percentage of X and Y Sperm in the Upper and Lower Layer of dsRNA-Treated Mouse Sperm

Sperm were incubated in an HTF medium containing 0.3 μM of dsRNA-40 or dsRNA-DR at 37 °C for 30 min. Subsequently, DNA was extracted from 350 μL of culture fluid collected from the upper layer, and the X/Y ratio of spermatozoa was determined by quantitative PCR.

The qPCR primers were designed to detect X and Y mouse sperm using the following sequences: Akap4-Forward: GGTAGAAGTAGCCCACGCAAACTC, Akap4-Reverse: CATTGAGGAGCCAGTTGAGGACAC (X sperm, PCR product size 134 bp); Ddx3y-Forward: TCAGAAAAGCGTAACAACTGGG, Ddx3y-Reverse: AGGACAACTTGTGAGGGGAGC (Y sperm, PCR product size 161 bp); Gapdh-Forward: ATCTGAAAGACAAGAAACAGGGG, Gapdh-Reverse: TTGTGGTACGTGCATAGCTGA (Internal reference gene, PCR product size 148 bp). PCR was conducted using a QuantStudio 7 Flex Real-Time PCR System (Applied Biosystems, Waltham, MA, USA) at 95 °C for 2 min, followed by 40 cycles consisting of denaturation at 95 °C for 15 s, annealing at 58.5 °C for 30 s, and extension at 72 °C for 20 s.

The Power SYBR Green master mix (Thermo Fisher) was used according to the manufacturer’s instructions. Each primer was confirmed to produce a single, specific PCR product through a melting curve program.

### 4.8. Identification of the Gender of IVF Embryos Generated by the Upper Layer of dsRNA-Treated Mouse Sperm

Sperm were incubated in an HTF medium containing 0.3 μM of dsRNA-40 or dsRNA-DR at 37 °C for 30 min. Then, 350 μL of sperm from the upper layer was collected and transferred to a new centrifuge tube. After centrifuging at 700 g for 4 min, the supernatant was discarded. The sperm were washed twice with 400 μL of overnight-equilibrated HTF medium and then resuspended in 50 μL of HTF medium and placed in the incubator.

Each 8-week-old female mouse was injected with 10 IU of PMSG at 18:00, followed by an injection of 10 IU of hCG 48 h later. After 14 h from the hCG injection, cumulus–oocyte complexes (COC) were collected from the female mouse’s oviduct. For each in vitro fertilization (IVF) experiment, 25 COCs were placed in 50 μL of HTF culture medium.

Five microliters of resuspended sperm were added to the fertilization drop, and the sperm density was adjusted to 1–5 × 10^6^/mL. Six hours after fertilization, the number of pronuclei in the oocyte was assessed to evaluate fertilization, followed by further culture in KSOM for embryo development analysis.

Some embryos were collected 4.5 days later, and PCR was employed for sexuality identification. The PCR primers were created to detect both Y chromosome and autosomes by following the given sequences: Rps18-Forward: TCTGTGGAGCGATGGAGTGTGA, Rps18-Reverse: GGCAGTGATGGCGAAGGCTATT (autosomes, PCR product size of 431 bp); Ddx3y-Forward: TGGACCAGCAAGTAAGTTGAACCT, Ddx3y-Reverse: GCCATTATGCGAAGCCTGTAGAGA (Y chromosome, PCR product size 231 bp). Each blastocyst was transferred to a 0.2 mL microcentrifuge tube containing 2 μL of KSOM embryo culture medium and subjected to PCR amplification using the direct PCR amplification kit (Sangon Biotech) as per the manufacturer’s instructions. ProFlex 3 × 32 well PCR System 9700 (Applied Biosystems, USA) was used with 1 min incubation at 98 °C, followed by 40 cycles of denaturation at 98 °C for 5 s, annealing at 64 °C for 5 s, and extension at 72 °C for 20 s. The PCR products were then visualized on 2% (*w/v*) agarose gels.

### 4.9. Statistical Analysis

All data are presented as the mean ± standard error from at least three independent experiments. Before statistical analysis, all data were tested for homogeneity of variance. Then, Student’s *t*-test or one-way ANOVA was used to evaluate the statistical significance between the two groups of data. Statistical analyses were conducted using GraphPad Prism 9.0 (GraphPad Software, San Diego, CA, USA). Data were subjected to one-way analysis of variance (ANOVA) and two-way ANOVA. Values with *p* < 0.05 were considered statistically significant.

## 5. Conclusions

TLR7/8 ligands dsRNA-40 and dsRNA-DR are effective in the separation of mouse X and Y sperm by the specific inhibition of X sperm motility by reducing its ATP level and mitochondrial activity. The use of a dsRNA-40- or dsRNA-DR-preselected upper layer of sperm for IVF produces embryos with a sex ratio skewed toward males. The dsRNA-40 and dsRNA-DR treatment of sperm is a potential method for mammalian animal gender control.

## Data Availability

The data that support the findings of this study are available on request from the corresponding author (Z.L.), upon reasonable request.

## Supplementary Materials

The following supporting information can be downloaded at: https://www.mdpi.com/article/10.3390/molecules29010262/s1, Figure S1: Technical Diagram of Mouse X/Y Sperm Isolation Method using dsRNA. Mouse spermatozoa were resuspended in 1 mL HTF containing 0.3 µM dsRNA and incubated at 37 °C for 30 minutes. Spermatozoa from the upper layer (Y spermatozoa) and lower layer (X spermatozoa) were then analyzed.
