# Supplementary material for: Gender Control of Mouse Embryos by Activation of TLR7/8 on X Sperm via Ligands dsRNA-40 and dsRNA-DR
Source: Molecules. 2024 Jan 4;29(1):262. doi: 10.3390/molecules29010262 (PMC10780660; doi:10.3390/molecules29010262)
Supplement: Supplementary file 1 [file molecules-29-00262-s001.zip › molecules-2782989-supplementary.pdf]

The procedure for sperm collection is as follows:

1. The night before the experiment, place the HTF semen in a 37°C CO2 incubator for overnight equilibration.

2. In the cell room's laminar flow hood, pipette 0.1ml of the overnight equilibrated HTF solution onto a 35mm cell culture dish to form a droplet. Cover with oil and place it in the CO2 incubator for later use. In the laminar flow hood, pipette 0.5ml of the overnight equilibrated HTF solution into a 1.5ml centrifuge tube. Retrieve it from the pass-through window and place it in a 37°C metal bath. After euthanizing the mouse, use ophthalmic scissors and forceps to extract the caudal epididymis and part of the vas deferens. Place them in the centrifuge tube containing HTF solution, pass through the pass-through window into the cell room, and, using two 1ml syringes, transfer the caudal epididymis of a single mouse into the prepared 35mm dish.

3. Holding a 1ml syringe in each hand, use the left hand to immobilize the caudal epididymis, and with the right hand, lightly puncture it multiple times with the needle of the syringe to allow some sperm to escape. Then, with coordinated movements of both hands, squeeze out the semen from the vas deferens. Finally, puncture the caudal epididymis with the needle to facilitate the release of more sperm. Incubate for 10 minutes at 37°C in a CO2 incubator. Using a syringe, pick up the caudal epididymis, simultaneously aspirating 900μl of overnight equilibrated HTF solution into a labeled 1.5ml centrifuge tube.

4. Aspirate 400μl of mineral oil with sperm-HTF solution into a 1.5ml centrifuge tube, centrifuge for 2 seconds using a mini centrifuge, aspirate the bottom 100μl of sperm-HTF solution, transfer it into 900μl HTF solution, and gently pipette four times with a pipetting gun to resuspend the sperm in the HTF solution. Place in the CO2 incubator.

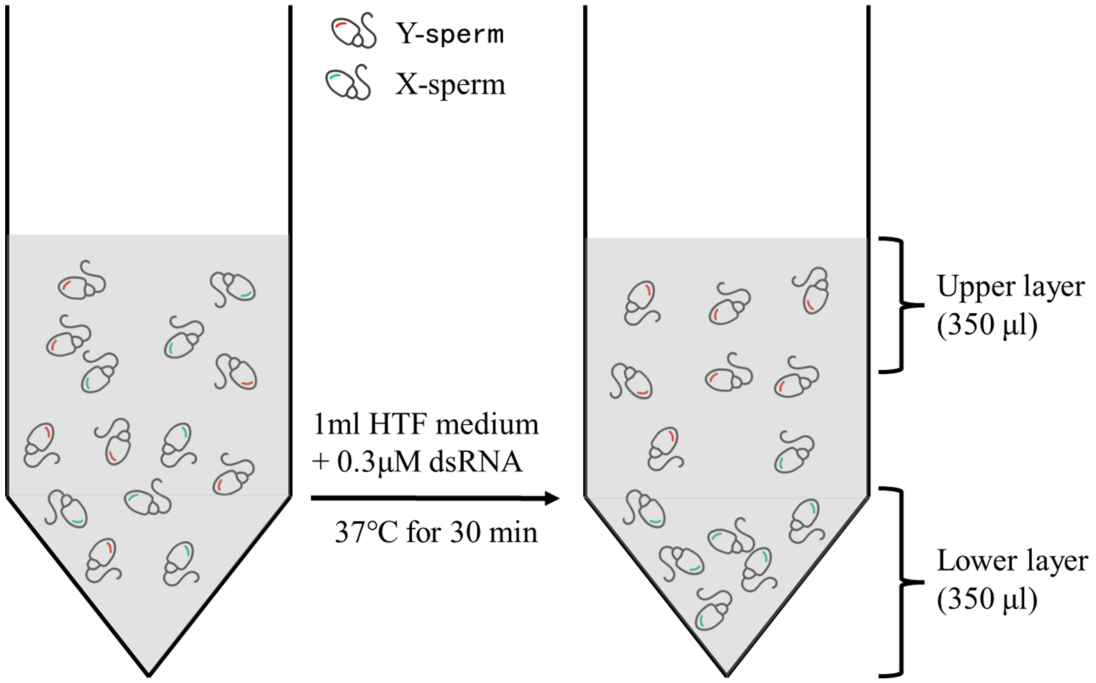

Figure S1. Technical Diagram of Mouse X/Y Sperm Isolation Method using dsRNA. Mouse spermatozoa were resuspended in 1 ml HTF containing 0.3 µM dsRNA and incubated at 37°C for 30 minutes. Spermatozoa from the upper layer (Y spermatozoa) and lower layer (X spermatozoa) were then analyzed.
